# Effect of parboiling conditions on zinc and iron retention in biofortified and non‐biofortified milled rice

**DOI:** 10.1002/jsfa.11379

**Published:** 2021-07-05

**Authors:** Victor Taleon, Md Zakiul Hasan, Roelinda Jongstra, Rita Wegmüller, Md Khairul Bashar

**Affiliations:** ^1^ HarvestPlus, c/o International Food Policy Research Institute (IFPRI) Washington DC USA; ^2^ HarvestPlus, c/o International Food Policy Research Institute Dhaka Bangladesh; ^3^ Laboratory of Human Nutrition, Department of Health Sciences and Technology, Institute of Food, Nutrition and Health ETH Zürich Zürich Switzerland; ^4^ HarvestPlus, c/o Alliance Bioversity‐CIAT Dhaka Bangladesh

**Keywords:** biofortification, degree of milling, parboiling, zinc rice, zinc retention

## Abstract

**BACKGROUND:**

Zinc‐biofortified rice could contribute to zinc intake in deficient populations, but processing it into parboiled rice could affect this potential benefit. Zinc and iron true retention (TR) in milled rice produced under conditions resembling household and commercial parboiled methods was evaluated. Zinc and iron TR in milled rice obtained from biofortified and non‐biofortified rice subjected to different soaking temperatures during parboiling was also evaluated.

**RESULTS:**

Conditions resembling commercial parboiling methods resulted in 52.2–59.7% zinc TR and 55.4–79.1% iron TR, whereas those used for household parboiling resulted in 70.7–79.6% zinc TR and 78.2–119.8% iron TR. Zinc TR in milled (8–16% bran removal) biofortified and non‐biofortified parboiled rice was 50.6–66.8% when soaking rough rice at 20 °C and 29.9–56.0% when soaking rough rice at 65 °C; both had lower zinc TR than non‐parboiled rice (58.0–80.6%). Iron TR was generally similar between milled non‐parboiled and parboiled rice (26.2–67.6%) and between parboiled biofortified and non‐biofortified milled rice.

**CONCLUSION:**

Parboiling conditions used to obtain milled rice targeted for own household consumption resulted in higher zinc and iron TR compared to parboiling conditions used for milled rice targeted for markets. More zinc from the inner endosperm moved towards the outer layers at high soaking temperature, resulting in lower zinc TR for milled parboiled rice soaked in hotter water. Parboiled rice soaked at temperatures used in households could provide more zinc to diets compared to rice soaked in hotter water commonly used in large rice mills, especially when rice is extensively milled. © 2021 The Authors. *Journal of The Science of Food and Agriculture* published by John Wiley & Sons Ltd on behalf of Society of Chemical Industry.

## INTRODUCTION

Zinc deficiency in South Asia exceeds 25%, mainly due to reliance on staple foods with low zinc content such as rice and wheat.^1^ Moderate zinc deficiency can result in clinical problems including growth retardation, cognitive impairment, cell‐mediated immune dysfunctions and lack of appetite.^2^ Rice varieties with zinc concentration up to 12 μg g^−1^ higher than in commercial varieties are being developed through biofortification to improve the daily zinc intake of deficient populations. Yet the additional zinc in biofortified rice could be reduced due to parboiling.^3^ Parboiling is a processing technique used to decrease the amount of broken rice during milling and reduce stickiness of cooked rice.^4^

In Bangladesh and India – the countries that produce the most parboiled rice and where biofortified rice is available – parboiling is done in households, small and medium commercial mills (SMED) and large mills called ‘automated rice mills’ (ARM).^3^, ^4^, ^5^, ^6^ Parboiling is done usually by soaking, steaming and drying rough rice. Rough rice parboiled in households or in SMED is commonly soaked at room temperature for several hours (up to 48 h), whereas in ARM it is soaked in hot water to accelerate water absorption (60**–**80 °C for 4**–**8 h). In SMED and ARM the soaked rough rice is heated by injecting steam into a metal bin where the grain is placed, whereas in households the soaked rough rice is heated in a pot partially filled with water, which provides steam to the grain above the water layer and boils the grain below it.^6^, ^7^, ^8^, ^9^

Milled parboiled rice usually contains more B vitamins, total minerals, potassium and phosphorous compared to milled non‐parboiled rice, because these nutrients migrate from the outer layers into the endosperm during parboiling. Hence parboiled rice is considered as more nutritious than non‐parboiled rice.^10^, ^11^, ^12^ However, lower zinc concentration has been reported in milled parboiled rice compared to milled non‐parboiled rice from markets in Bangladesh and Brazil.^12^, ^13^, ^14^ Taleon *et al*.^3^ reported 21**–**25% higher zinc losses during milling of parboiled rice (7.5**–**10.0% bran removal and soaked at 20 °C) compared to non‐parboiled rice. Up to 44% higher zinc losses were found by Denardin *et al*.^15^ in milled parboiled rice obtained from rough rice soaked in hot water (>60 °C) compared to milled non‐parboiled rice. Large and variable losses of iron (25**–**77%) have also been reported in parboiled rice.^3^, ^9^ The wide range of zinc and iron losses in milled parboiled rice reported in these previous studies suggests that parboiling causes a reduction in the final zinc and iron concentration, but it is not clear which parboiling factors affect their concentration the most.

Considering the diverse parboiling conditions, milling intensity and the highly variable zinc and iron retention in parboiled rice from previous studies, it is expected that zinc and iron losses during parboiling vary widely in South Asia due to the diverse parboiling methods used. It is important to identify parboiling conditions that result in higher zinc and iron concentration in biofortified and non‐biofortified rice. Therefore, the zinc and iron true retention (TR) in non‐biofortified milled rice obtained from rough rice subjected to parboiling conditions resembling household and commercial parboiled methods using different water types was evaluated in the first part of the study. Furthermore, zinc and iron TR in milled rice obtained from biofortified (high zinc content) and non‐biofortified rough rice subjected to different soaking temperatures during parboiling was evaluated in the second part of the study.

## METHODOLOGY

### Sources of grain and water

Rough rice of zinc‐biofortified rice variety BRRI dhan42 was harvested during Aman (rainfed) 2016 at Jessore, Bangladesh, and zinc‐biofortified variety BRRI dhan64 and non‐biofortified variety BRRI dhan28 were harvested during Boro (irrigated) 2015**–**2016 at different fields in Bogura, Bangladesh. After harvesting, rough rice was sundried to a moisture content of 12**–**14%. Two water types were used for soaking: commercial bottled water with low hardness (SOFT) and water from the local municipal system (HARD) from Bogura, Bangladesh. SOFT had <0.1 mg L^−1^ of zinc and <0.1 mg L^−1^ of iron and hardness <10 mg L^−1^ CaCO_3_ equivalents, whereas HARD had 0.3 mg L^−1^ of zinc, 3.6 mg L^−1^ of iron and hardness of 133 mg L^−1^ CaCO_3_ equivalents. The amount of water added for soaking was 1.3 times the weight of rough grain in all soaking batches to minimize the effect of using different quantities of rough rice during soaking.

### Parboiling methods

In the first part of the study, the parboiling conditions evaluated resembled those used in households (BOIL), small commercial parboiling facilities when parboiling grain for clients that will use it for household consumption (LIM), SMED facilities for rice to be sold in markets (COM1) and ARM facilities (COM2). To resemble the soaking conditions used in the BOIL, LIM and COM1 methods, 600 g of raw rough rice variety BRRI dhan28 was soaked for 24 h at 20 °C in either SOFT or HARD. To simulate the soaking conditions used in the COM2 method, 200 g of raw rough rice was soaked in SOFT or HARD at 65 °C. Rice soaked at 20 °C was placed in a room with the air conditioner set at 20 °C, whereas rice soaked at 65 °C was placed in a water bath (Supporting Information, Fig. SS1). For the second part of the study, to evaluate the effect of soaking water temperature and variety on zinc and iron TR, 3400 g of raw rough rice of each variety was soaked in SOFT at 20 °C (24 h) and at 65 °C (4 h) and were identified as S20, and S65, respectively (Supporting Information, Fig. SS2). Only SOFT was used when evaluating the effect of soaking water temperature and variety on zinc and iron TR, to control for any potential confounding effect caused by water hardness and water zinc and iron concentration. All soaking batches were produced in triplicate.

After soaking, batches of rough rice for BOIL, LIM, COM1 and COM2 parboiling methods were steamed with one of three steaming conditions: boiling, limited steaming or complete steaming (Supporting Information, Fig. SS1). For BOIL, steaming was completed by placing 200 g of rough rice soaked at 20 °C inside an aluminum pot containing boiling water using a rice‐to‐water ratio of 2:1 for 13 min. The resulting rough rice was identified as SOFTBOIL and HARDBOIL for rice processed with SOFT and HARD, respectively. For LIM and COM1, 200 g of rough rice soaked at 20 °C was steamed inside an aluminum pot with 5 mm openings that was placed on top of a larger pot containing boiling water. Steaming time for LIM was 7 min and for COM1 was 14 min. Rough rice batches for LIM soaked with SOFT and HARD were identified as SOFTLIM and HARDLIM, respectively. Similarly, batches of COM1 soaked with SOFT and HARD were identified as SOFTCOM1 and HARDCOM1, respectively. For COM2, the batches of 200 g of rough rice soaked at 65 °C were treated with complete steaming (14 min) to resemble the parboiling conditions used in ARM facilities and the resulting steamed batches were named SOFTCOM2 when soaked with SOFT and HARDCOM2 when soaked with HARD (Supporting Information, Fig. SS1). To evaluate the effect of soaking temperatures and varieties, each batch of soaked rough rice S20 and S65 was steamed (14 min) to obtain PB20 and PB65 (Supporting Information, Fig. SS2). Non‐parboiled grains from each variety were used as controls and identified as NPB. All steamed rice batches were sun‐dried until their moisture reached 18**–**20% and tempered overnight. After tempering, drying continued until moisture reached 12**–**14%.

### Milling

Rough rice parboiled with BOIL, LIM, COM1 and COM2 was dehulled in a rubber sheller machine (Miltec, Hong Kong) to obtain brown rice. Broken brown rice was removed after dehulling. To obtain milled rice, 57.00 ± 0.04 g of unbroken (head) brown rice subsample from each batch of parboiled rice was milled (28.50 g at a time) to remove the bran (pericarp + most of the embryo + most of the aleurone + some endosperm) using a Miltec lab rice mill (Miltec, Hong Kong). The percentage of bran removed, also called degrees of milling (DOM), was 7.5% ± 0.5% for the batches parboiled using the household and commercial methods (Supporting Information, Fig. SS1). DOM was estimated by measuring the weight of the dehulled grain before and after milling. Milling pretests were completed for each batch of parboiled brown rice to ensure that each milling batch used for analysis reached the target DOM.

For PB20, PB65 and their NPB controls, rough rice samples were dehulled as described previously. For milling, 28.50 ± 0.02 g of head brown rice of each parboiled and non‐parboiled batch was milled for 5**–**10 s to obtain milled rice with 1.0% ± 0.5% DOM. Milling pretests were done to reach the desired target DOM for each batch analyzed. Broken and head rice was separated and only the head rice was used for zinc and iron analysis using a non‐destructive method. After being analyzed for zinc and iron, the same milled samples were further milled for 5**–**40 s to obtain the next DOM level (1.0% ± 0.5% DOM increase), and after separating broken grain the samples were reanalyzed for zinc and iron. The milling, classification and zinc and iron measurements were repeated 14 more times to achieve a final DOM of 16% (Supporting Information, Fig. SS2).

### Zinc and iron analysis

Zinc and iron concentration in all head rice produced using the household and commercial parboiling methods were analyzed by flame atomic absorption spectroscopy (FAAS) using an Agilent AA240FS spectrometer (Agilent Technologies, Palo Alto, CA, USA). Zinc and iron were extracted from kernels (200 ± 5 mg) after mineralization in HNO_3_ by microwave‐assisted digestion (MLS TurboWave, MLS GmbH, Leutkirch, Germany). All materials were acid washed. All samples were analyzed in triplicate. A rice flour Standard Reference Material (1568b, NIST, Gaithersburg, MD, USA) was analyzed in duplicate, with every measurement run to monitor accuracy as well as duplicated standard blanks. Since the analysis of minerals by FAAS is a destructive method, it was not possible to analyze the zinc and iron content with this method at different DOM in the same sample. For this reason, all head rice samples used to evaluate the soaking water temperature and variety effects were analyzed by a non‐destructive energy‐dispersive X‐ray fluorescence spectrometry (XRF) method using a Bruker S2 RANGER spectrometer (Bruker AXS GmbH, Karlsruhe, Germany). For XRF analysis, 12 ± 2 g of cleaned head rice sample was placed into a plastic cup, which was then put on the sample tray of the XRF spectrometer for analysis, as described by Paltridge *et al*.^16^

### True zinc and iron retention

True retention (TR) percentage values were used to determine the total proportion of zinc lost during processing, considering the dry matter losses of milling. For all samples, TR percentage was calculated as follows:

$$\mathrm{TR}\%=\frac{\text{zinc content}\;\mathrm{per}\;g\;\text{processed rice}\times g\;\text{rice after processing}}{\text{zinc content}\;\mathrm{per}\;g\;\text{brown rice before processing}\times g\;\text{brown rice before processing}}$$

### Contribution to zinc estimated average requirement (EAR)

To estimate the contribution of biofortified and non‐biofortified rice to zinc intake in target populations, per capita daily raw rice consumption of 420 g for women of child‐bearing age and 134 g for children of preschool age from Bangladesh was used as a reference for maximum contribution.^17^ Zinc TR values for biofortified variety BRRI dhan64 and non‐biofortified variety BRRI dhan28 processed as parboiled and non‐parboiled rice and milled at different DOM (0, 8, and 16%) were used to calculate the contribution of rice to the EAR of zinc. An EAR of 10.0 mg for pregnant women and 4.0 mg for children aged 4–8 years who both use an unrefined cereal based diet was used as a reference.^18^

### Data analysis

The effects of water type and common parboiling methods on zinc and iron TR were evaluated using a two‐way analysis of variance (ANOVA). Similarly, the variety and soaking temperature effects on zinc and iron TR were evaluated using a two‐way ANOVA (at 8% and 16% DOM). Means separations for water type, common parboiling methods, variety and soaking temperature were completed using Duncan's multiple range test. Differences between means were considered significant at *P* < 0.05. Analysis was done using SAS 9.4 (SAS Institute, Cary, NC, USA).

## RESULTS AND DISCUSSION

### Zinc and iron TR in rice parboiled with BOIL, LIM, COM1 and COM2

Zinc and iron concentration in non‐parboiled brown rice variety BRRI dhan28 was 20.7 ± 1.2 and 10.6 ± 0.5 μg g^−1^, respectively. After parboiling using conditions resembling those commonly used in households and commercial facilities in South Asia, zinc TR in brown rice was 96.4–105.4%, while in milled rice (7.5% DOM) it was 52.2–79.6% (Table 1). The zinc TR in milled parboiled rice was more variable than that of brown parboiled rice, and also more variable than the 61–76% zinc retention reported by Mayer *et al*.^12^ in 33 diverse samples of milled parboiled rice obtained from Bangladesh with unknown DOM. Iron TR in brown parboiled rice was 100.8–114.2%, whereas in milled parboiled rice it was 55.4–119.8% (Table 1). The large variation in zinc and iron TR in milled rice was mainly due to the parboiling method but not the water type used for parboiling, as detailed below.

**Table 1:** Zinc and iron concentrations (μg g^−1^) and true retention (TR%) of brown (DOM0) and milled (DOM7.5) rice variety BRRI dhan28 parboiled using two sources of water (SOFT and HARD) and four parboiling methods (BOIL, LIM, COM1 and COM2)

Process^†^	Brown rice (DOM0)				Milled rice (DOM7.5)			
	Zinc (μg g^−1^)	Zinc (TR%)	Iron (μg g^−1^)	Iron (TR%)	Zinc (μg g^−1^)	Zinc (TR%)	Iron (μg g^−1^)	Iron (TR%)
SOFTBOIL	21.8 ± 0.8a	105.4 ± 3.6a	11.9 ± 0.9a	112.5 ± 8.5a	16.7 ± 0.3ab	74.3 ± 1.4ab	9.8 ± 1.1b	85.1 ± 9.3b
HARDBOIL	21.2 ± 0.7abc	102.5 ± 3.2abc	12.1 ± 0.2a	114.2 ± 1.9a	15.9 ± 0.8b	70.7 ± 3.8b	13.8 ± 1.4a	119.8 ± 12.0a
SOFTLIM	19.9 ± 0.4c	96.4 ± 1.8c	12.0 ± 1.2a	113.4 ± 11.3a	17.5 ± 0.1a	77.9 ± 0.3a	9.0 ± 1.1bc	78.2 ± 9.3bc
HARDLIM	21.1 ± 0.5abc	102.1 ± 2.6abc	11.8 ± 0.1a	111.1 ± 0.7a	17.9 ± 1.0a	79.6 ± 4.2a	9.1 ± 1.1bc	79.2 ± 9.6bc
SOFTCOM1	20.9 ± 1.0abc	101.1 ± 4.8abc	11.8 ± 0.1a	111.5 ± 1.3a	13.4 ± 1.0c	59.7 ± 4.6c	9.1 ± 0.8bc	79.1 ± 6.6bc
HARDCOM1	20.4 ± 1.2abc	98.4 ± 5.7abc	10.7 ± 0.2b	100.8 ± 1.5b	13.3 ± 0.7c	59.2 ± 3.1c	6.4 ± 0.7d	55.4 ± 5.8d
SOFTCOM2	21.7 ± 1.5ab	104.7 ± 7.4ab	11.4 ± 0.6ab	107.8 ± 5.9ab	12.4 ± 0.3cd	55.2 ± 1.5cd	7.9 ± 0.7cd	68.8 ± 6.0cd
HARDCOM2	20.1 ± 0.3bc	97.1 ± 1.5bc	11.3 ± 0.2ab	106.8 ± 2.0ab	11.7 ± 0.7d	52.2 ± 2.9d	6.5 ± 0.7d	56.7 ± 5.8d

Mean ± standard deviation of three parboiling batch repetitions. Different lower‐case superscript letters indicate significant differences within each column (*P* < 0.05). DOM7.5, 7.5% degrees of milling.

^†^ SOFTBOIL and HARDBOIL = soaked at 20 °C and boiled for 13 min; SOFTLIM and HARDLIM = soaked at 20 °C and 7 min steam; SOFTCOM1 and HARDCOM1 = soaked at 20 °C and 14 min steam; SOFTCOM2 and HARDCOM2 = soaked at 65 °C and 14 min steam. Processes codes containing SOFT indicate that samples were soaked using commercial bottled water; codes containing HARD indicate that samples were soaked using water from the local municipal system in Bogura, Bangladesh.

**Figure 1:**
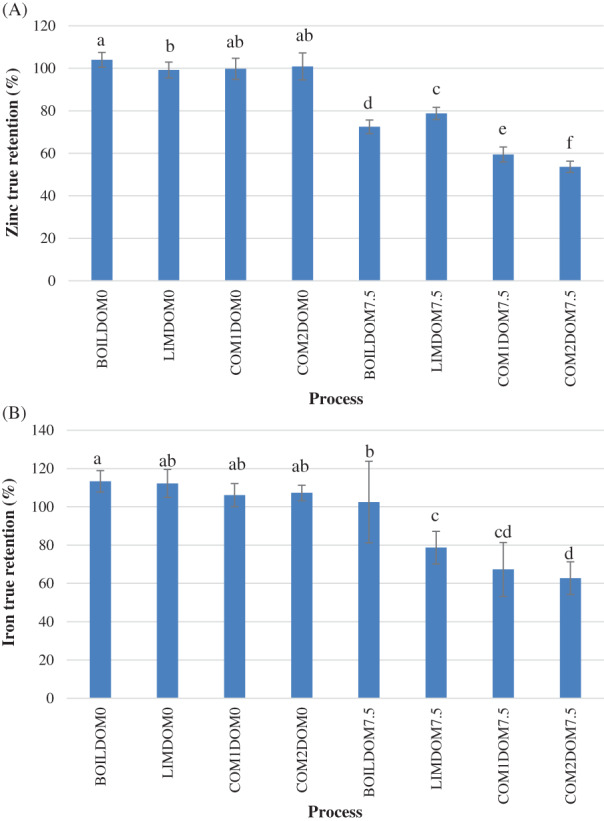
Zinc (A) and iron (B) true retention in rice parboiled with different methods (BOIL = soaked at 20 °C and boiled 13 min; LIM = soaked at 20 °C and steamed for 7 min; COM1 = soaked at 20 °C and steamed for 14 min; COM2 = soaked at 65 °C and steamed for 14 min) and milled at different levels (DOM0, brown rice; DOM7.5, 7.5% degrees of milling). Mean ± standard deviation of two soaking water types and three processing repetitions. Bars with different letters within each processing type are statistically different (*P* < 0.05).

Zinc TR in brown parboiled rice produced with rough rice soaked in SOFT and HARD was 96.4–105.4% and 97.1–102.5%, respectively. Iron TR of brown parboiled rice was 107.8–113.4% when using SOFT and 100.8–114.2% when using HARD. In general, all milled parboiled rice had lower zinc and iron TR than their respective parboiled brown rice (Fig. 1(A,B)). TR higher than 100% in brown grain generally found for iron was probably due to the mobilization of iron from the husk to the kernel. Compared to brown rice, higher concentrations of iron but not zinc have been found in husk,^19^ which could facilitate the mobilization of iron from the husk to the brown rice. The high concentration of iron in the husk could also be the reason for the broader range of TR values obtained for iron, as observed in previous studies.^3^, ^15^

In general, no significant differences in zinc and iron TR were found between milled parboiled rice produced with SOFT and HARD. After milling, zinc TR was 55.2–77.9% when rice was parboiled with SOFT and 52.2–79.6% when parboiled with HARD (Table 1). Iron TR of rice soaked in SOFT and HARD was 68.8–85.1% and 55.4–119.8%, respectively (Table 1). An increase in zinc TR when rice was soaked in water with high zinc concentration (50–400 mg L^−1^) for fortification purposes was observed in previous studies.^20^, ^21^ However, zinc concentration when using HARD was lower than that used in fortification studies and was not able to increase zinc TR of parboiled samples. Water with hardness up to 133 mg L^−1^ CaCO_3_ equivalents did not affect the TR, suggesting that other minerals present in soft to moderately hard water do not affect zinc and iron TR in milled parboiled rice. Considering that the water type did not affect zinc and iron TR, but the variability in zinc and iron TR for parboiled rice milled at 7.5% DOM was large, the effect of each parboiling method was evaluated.

Significant differences in zinc and iron TR were found between milled rice (7.5% DOM) parboiled under conditions resembling household and commercial methods. Rice processed with BOIL and LIM had higher zinc TR (70.7**–**79.6%) than rice processed with COM1 and COM2 (52.2**–**59.7%) (Table 1). Zinc TR for BOIL and LIM was also higher than TR (56.1**–**64.9%) reported by Taleon *et al*. for milled parboiled rice using a parboiled method similar to COM1.^3^ For iron, milled rice subject to BOIL had higher TR (85.1**–**119.8%) than rice subject to COM2 (56.7**–**68.8%) but not always higher than rice subject to LIM and COM1 (Table 1). When comparing the main differences in parboiling conditions between BOIL and COM1, it appeared that the use of direct steam in COM1 exacerbated the zinc losses once parboiled grain was milled. Nevertheless, the negative effect of direct steam was observed only when it was used for a long period, as suggested by the lower zinc TR obtained with COM1 compared to LIM (Table 1). The soaking temperature could also be an important reason for the low zinc TR in rice parboiled with COM2, considering that rice parboiled with this method had the lowest zinc TR and was the only one soaked in water at high temperature.Since BOIL and LIM are used to parboil rice to be consumed directly by households without passing through markets, and COM1 and COM2 are used when parboiled rice is produced to be sold in markets, it is suggested that milled parboiled rice produced and consumed in households will generally have higher zinc retention than parboiled rice sold in markets. To better understand the low zinc and iron TR values in rice parboiled produced with COM1 and COM2, the soaking conditions that resembled those usually found in SMED and ARM were further evaluated in three rice varieties at different DOM (8% and 16%).

**Figure 2:**
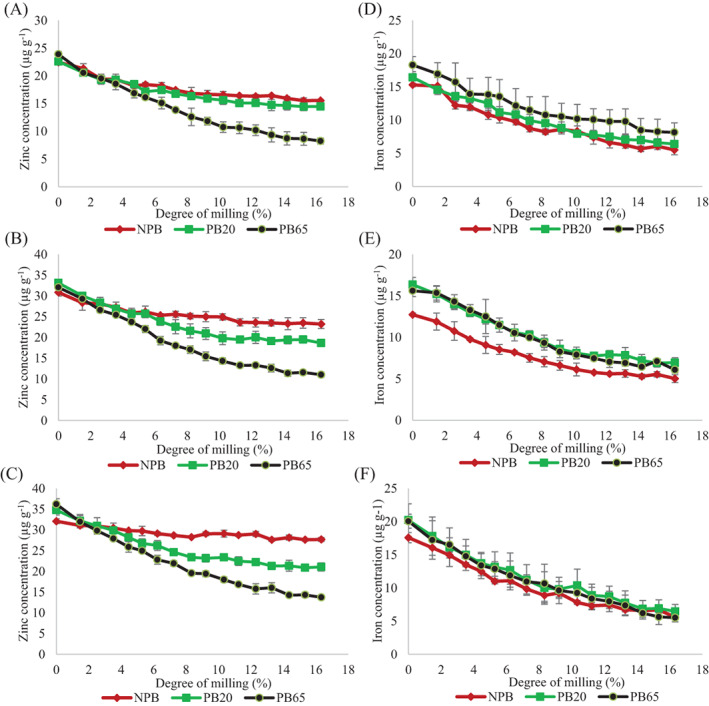
Zinc (A–C) and iron (D–F) concentration in rice milled at different degrees (0–16%) processed as: non‐parboiled (NPB), parboiled rice soaked at 20 °C for 24 h (PB20) and parboiled rice soaked at 65 °C for 4 h (PB65) for variety BRRI dhan28 (A,D), BRRI Dhan42 (B,E) and BRRI dhan64 (C,F). Mean ± standard deviation of three parboiling repetitions.

In South Asia, farmers generally request millers to mill their rice at low DOM when the rice is expected to be used at their own household. However, millers and farmers prefer higher DOM for rice that will be distributed to markets because such rice can command a higher price because of its whiter color, which is attractive to consumers.^11^ DOM of 7–8% could be considered optimal from a milling yield optimization perspective, because less broken rice is produced and the oil content is below the maximum acceptable for shelf life stability (<1%). Lamberts *et al*.^22^ reported that milling increased whiteness of the rice due to removal of pericarp and germ (8% DOM) and outer endosperm (9–15% DOM), noting that whiteness did not increase after further milling. Considering this, the zinc and iron TR was determined for non‐parboiled and parboiled rice of varieties with high and low initial zinc concentration milled up to 16% DOM (Fig. 2(A–F)).

### Effect of soaking temperature on zinc and iron TR

#### Zinc and iron TR in milled non‐parboiled rice

Zinc concentration in NPB brown rice (DOM0) of the non‐biofortified variety BRRI dhan28 was 22.5 ± 0.1 μg g^−1^, whereas for the zinc biofortified varieties BRRI dhan42 and BRRI dhan64 it was 30.9 ± 0.8 μg g^−1^ and 32.1 ± 0.6 μg g^−1^, respectively (Table 2). Biofortified varieties had 37**–**43% higher zinc concentration than the non‐biofortified variety, which was expected because these biofortified varieties were bred to have higher zinc concentration compared to other commercial varieties. The non‐biofortified variety had a similar zinc concentration to that of 139 samples of commercial brown rice from Bangladesh (18.6**–**20.8 μg g^−1^).^12^ Iron concentration in NPB DOM0 was 15.3 ± 0.3 μg g^−1^ for BRRI dhan28, whereas for the biofortified varieties BRRI dhan42 and BRRI dhan64 it was 12.7 ± 0.2 μg g^−1^ and 17.6 ± 0.8 μg g^−1^, respectively (Table 2). Iron concentration was within the 10.1**–**26.4 μg g^−1^ previously reported for non‐parboiled brown rice.^23^ In an earlier study, a positive correlation was found between zinc and iron concentration in rice grain due to possible co‐localization of genes responsible for high zinc accumulation with genes for high iron accumulation,^24^ however, in this study iron concentration in zinc‐biofortified rice was not always higher than in non‐biofortified rice (Table 2).

**Table 2:** Zinc and iron concentration (μg g^−1^) and true retention (TR%) of brown (DOM0) and milled (DOM8 and DOM16) rice of three varieties after processed as non‐parboiled rice (NPB), parboiled rice soaked at 20 °C (PB20) and parboiled rice soaked at 65 °C (PB65)

Nutrient	Variety	Process^†^	Concentration (μg g^−1^) in DOM0	TR% in DOM0	Concentration (μg g^−1^) in DOM8	TR% in DOM8	Concentration (μg g^−1^) in DOM16	TR% in DOM16
Zinc	BRRI dhan28	NPB	22.5 ± 0.1e	100.0 ± 0.6c	16.9 ± 1.1f	69.1 ± 4.4bc	15.6 ± 0.1e	58.0 ± 0.6c
		PB20	22.6 ± 0.5e	100.5 ± 2.4c	16.3 ± 0.6f	66.7 ± 2.2c	14.5 ± 0.5ef	54.1 ± 2.1d
		PB65	23.9 ± 0.5e	106.4 ± 2.3b	12.6 ± 1.6g	51.5 ± 6.6d	8.3 ± 0.5h	30.7 ± 2.0g
	BRRI dhan42	NPB	30.9 ± 0.9d	100.0 ± 2.8c	25.2 ± 0.7b	74.8 ± 2.1ab	23.2 ± 1.1b	63.0 ± 2.8b
		PB20	33.1 ± 0.7bc	107.4 ± 2.3b	21.6 ± 1.7d	64.3 ± 5.1c	18.7 ± 0.4d	50.6 ± 1.1e
		PB65	32.1 ± 2.0cd	103.9 ± 6.4bc	17.1 ± 0.1f	50.8 ± 0.3d	11.0 ± 0.8g	29.9 ± 2.0g
	BRRI dhan64	NPB	32.1 ± 0.6cd	100.0 ± 1.8c	28.3 ± 0.1a	80.6 ± 0.4a	27.7 ± 0.5a	72.3 ± 1.2a
		PB20	34.8 ± 1.2ab	108.3 ± 3.7b	23.4 ± 0.4c	66.8 ± 1.4c	21.1 ± 1.0c	55.0 ± 2.7cd
		PB65	36.2 ± 1.3a	112.8 ± 4.1a	19.6 ± 0.4e	56.0 ± 1.2d	13.7 ± 0.4f	35.7 ± 1.1f
								
Iron	BRRI dhan28	NPB	15.3 ± 0.3d	100.0 ± 1.8d	8.5 ± 0.4ab	51.3 ± 2.2ab	5.5 ± 0.7bc	30.1 ± 3.9cd
		PB20	16.4 ± 0.9bcd	107.4 ± 5.6cd	9.5 ± 0.8ab	57.1 ± 4.9ab	6.4 ± 0.5bc	35.1 ± 2.7bc
		PB65	18.3 ± 1.3ab	119.6 ± 8.3abc	10.8 ± 2.8a	64.6 ± 16.5ab	8.2 ± 1.4a	44.5 ± 7.8a
	BRRI dhan42	NPB	12.7 ± 0.2e	100.0 ± 1.6d	7.7 ± 0.6b	50.9 ± 4.5ab	5.0 ± 0.5c	33.0 ± 3.0bcd
		PB20	16.4 ± 0.9bcd	128.5 ± 6.9a	9.4 ± 0.9ab	67.6 ± 6.6a	6.9 ± 0.6ab	45.7 ± 4.0a
		PB65	15.6 ± 0.7cd	122.7 ± 5.6ab	9.3 ± 0.5ab	67.3 ± 4.0a	6.1 ± 0.4bc	40.0 ± 2.6ab
	BRRI dhan64	NPB	17.6 ± 0.8bc	100.0 ± 4.4d	8.9 ± 0.9ab	46.3 ± 4.9b	5.6 ± 0.2bc	26.7 ± 1.1d
		PB20	20.2 ± 2.5a	115.0 ± 14.0bc	10.0 ± 2.5ab	52.1 ± 12.7ab	6.5 ± 1.0bc	30.8 ± 5.0cd
		PB65	20.1 ± 1.1a	114.1 ± 6.0bc	10.7 ± 2.9a	55.7 ± 15.2ab	5.5 ± 0.6bc	26.2 ± 2.9d

Different letters indicate significant differences within columns for each nutrient (*P* < 0.05). Values are mean ± standard deviation of three processing repetitions. DOM8, rice milled at 8% degrees of milling; DOM16, rice milled at 16% degrees of milling.

^†^ NPB, non‐parboiled grain; PB20, soaked at 20 °C for 24 h; PB65, soaked at 65 °C for 4 h; TR, percentage of true retention.

**Figure 3:**
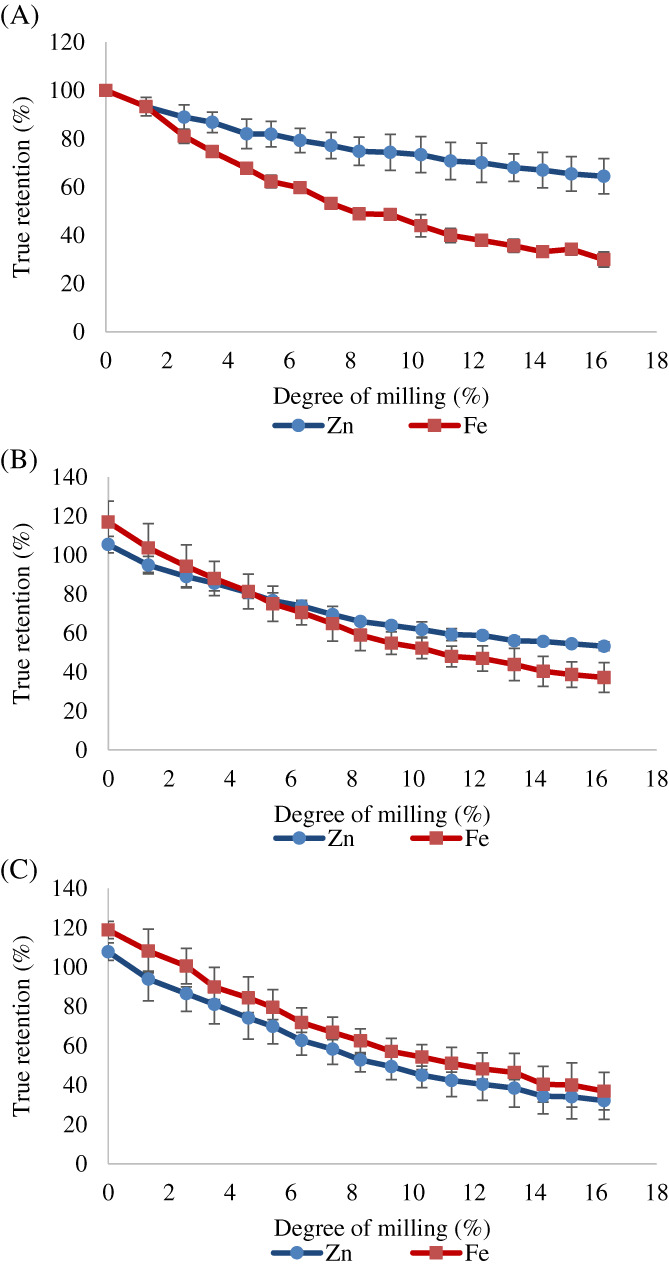
Zinc and iron true retention in rice milled at different degrees (0–16%) processed as: non‐parboiled (A), parboiled rice soaked at 20 °C for 24 h (B) and parboiled rice soaked at 65 °C for 4 h (C). Mean ± standard deviation of three varieties.

Zinc TR in NPB milled rice (DOM8) of the non‐biofortified variety BRRI dhan28 was 69.1%, similar to the 74.8% obtained in the biofortified variety BRRI dhan42 but lower than the 80.6% obtained with BRRI dhan64 (Table 2). Iron TR in NPB (DOM8) was 46.3–51.3% and no differences were found between varieties (Table 2). Zinc and iron TR in NPB rice with 16% DOM was 58.0–72.3% and 26.7–33.0%, respectively (Table 2). In general, NPB rice with 16% DOM had lower zinc and iron concentration than brown rice and rice milled with 8% DOM (Fig. 2(A–F)). However, zinc TR after milling was higher than iron TR for all genotypes combined (Fig. 3(A)). The low TR values found in milled biofortified and non‐biofortified non‐parboiled rice are in concordance with previous studies, which reported that rice bran contains higher zinc and iron concentration compared to the endosperm.^25^, ^26^ The higher Zn TR found in the biofortified variety BRRI dhan64 compared to the non‐biofortified variety BRRI dhan28 suggests that the additional zinc in this biofortified variety is more concentrated in the endosperm, similar to the findings of Taleon *et al*.^3^ when comparing biofortified and non‐biofortified rice. The zinc concentration decreased less (10.4%) compared to iron (18.8%) when removing the outer endosperm in non‐parboiled rice (from 8% to 16% DOM), similar to the trend observed when removing the outermost layers (from 0% to 8% DOM) (Fig. 3(A)), suggesting that the zinc in raw biofortified grain is more uniformly distributed compared to iron, as also observed by Lu *et al*.^19^ and Wang *et al*.^26^ in non‐biofortified rice.

#### Zinc and iron TR in brown and milled parboiled rice

After parboiling PB20 and PB65, zinc TR in brown rice was 100.5–112.8%, while iron TR was 107.4–128.5% (Table 2). TR higher than 100% indicated that zinc and iron moved from the husk to the grain during parboiling. However, zinc concentration in parboiled rice milled at 8% DOM was as low as 16.3 μg g^−1^ for PB20 and 12.6 μg g^−1^ for PB65, resulting in lower TR when rough rice was soaked at 65 °C (50.8–56.0%) compared to rough rice soaked at 20 °C (64.3–66.8%) (Table 2). Zinc TR in milled parboiled rice was lower than in non‐parboiled rice regardless of the soaking method, except for PB20 of rice variety BRRI dhan28 (Table 2). Iron TR in milled parboiled rice (8% DOM) was 52.1–67.6% TR (Table 2). Contrary to zinc, no differences were found for iron TR within PB20 and PB65 for each variety (Table 2).

When parboiled rice was milled to a higher degree (16% DOM), zinc concentration in PB20 was 14.5–21.1 μg g^−1^ and in PB65 was only 8.3–13.7 μg g^−1^, resulting in lower TR in rice soaked at 65 °C (29.9–35.7% TR) compared to 20 °C (50.6–55.0% TR) and non‐parboiled rice milled at the same DOM (Table 2). Regardless of the soaking temperature, zinc TR in parboiled rice milled at 16% DOM was lower than when milled at 8% DOM (Fig. 3(B,C)). Furthermore, iron TR in parboiled rice with 16% DOM was 26.2–45.7% (Table 2), which was lower than when milled at 8% DOM (Fig. 3(E,F)). In general, zinc in rice soaked at 65 °C was lost at a similar rate to iron during milling, in contrast to milled non‐parboiled and parboiled rice soaked at 20 °C, where zinc was lost at a lower rate than iron (Fig. 3(A–C)).

In general, the zinc that moved from the husk to the kernel during parboiling did not reach the endosperm (Fig. 2(A–C)). Furthermore, zinc from the inner endosperm of rice moved towards the outer layers of the kernel during parboiling, resulting in lower zinc concentration in milled parboiled rice, especially when rice was soaked at high temperature. Iron losses after milling parboiled rice were similar to non‐parboiled rice (Fig. 2(D–F)). The behavior of zinc and iron during parboiling contrasted with the higher accumulation of other important nutrients; for example, Juliano^27^ found that thiamine increased after parboiling rice, and Denardin *et al*.^15^ reported that magnesium, potassium and phosphorus concentrations were higher in milled parboiled rice compared to milled non‐parboiled rice.

Based on the zinc and iron concentrations found in milled parboiled rice, it is suggested that zinc but not iron migrated from the inner endosperm to the outer layers of the kernel during parboiling and was trapped in such layers, probably due to the formation of complexes with phytic acid. High accumulation of phytic acid in the outer layers of rice^3^, ^25^, ^26^ and higher affinity of phytic acid to zinc compared to iron^28^, ^29^, ^30^ could be the reason why zinc that moved from the endosperm remained in the bran layer. Oli *et al*.^31^ found a thicker layer of zinc in the outer structures of the kernel after soaking rice, especially in water at high temperature, whereas Hummel *et al*.^32^ found that beans with higher phytic acid content retained more zinc during soaking. Considering that the bioavailability of zinc is low when bound to phytic acid,^1^ the bioavailability of zinc in brown parboiled rice or parboiled rice with a low DOM should be studied to determine whether the zinc in these grains is as bioavailable as that present in non‐parboiled rice.

#### Contribution of parboiled rice to daily zinc intake

Considering the broad range of zinc TR obtained by using different parboiling and milling methods, the potential contribution to daily zinc EAR (4 mg for children and 10 mg for pregnant women)^18^ was calculated for relevant combinations of processing (Table 3). For children aged 4–8 years, biofortified rice variety BRRI Dhan64 would contribute 70–78% of the daily zinc requirement when consuming milled non‐parboiled rice. However, if rice is parboiled, the contribution of biofortified rice to the EAR would decrease to 35–65% after milling. The contribution of zinc‐biofortified rice to daily zinc EAR could be up to 97% if consumed as non‐parboiled brown rice, although the phytic acid of brown rice could limit its bioavailability.^33^ Furthermore, non‐biofortified rice variety BRRI Dhan28 could contribute 68% to daily zinc EAR when consumed as milled non‐parboiled rice and only 21% if consumed as parboiled rice with high DOM, as typically produced in medium‐ and large‐scale milling facilities. Similarly, for pregnant women, the contribution of zinc‐biofortified rice could cover 100% of their EAR when eaten as milled non‐parboiled but only 44–83% when eaten as milled parboiled (Table 3). Given the large effect of processing, the contribution of biofortified rice to daily zinc EAR% in deficient populations should be calculated based on whether the rice is consumed as parboiled or non‐parboiled, whether parboiling was done under household conditions (soaking at room temperature) or at large commercial milling facilities (soaking in hot water) and on the expected degree of milling preferred for each population.

**Table 3:** Potential contribution of biofortified rice variety BRRI Dhan64 and non‐biofortified rice variety BRRI Dhan28 to the zinc percentage estimated average requirement (EAR) based on zinc true retention values of non‐parboiled (NPB) and parboiled rice (PB20 and PB65)

Process^a^	Degree of milling (%)	Rice type	Zn milling retention (%)	Zn in milled rice (μg g^−1^)^b^	Zn daily intake for children (mg)	Zn daily intake for women (mg)	EAR for children (%)^c^	EAR for women (%)^c^
NPB	0	BF	100	32.1	3.9	12.4	97	124
	0	NBF	100	22.5	2.7	7.8	68	78
	8	BF	78	25.9	3.1	10.0	78	100
	8	NBF	69	15.5	1.9	5.4	47	54
	16	BF	68	23.2	2.8	9.0	70	90
	16	NBF	58	13.1	1.6	4.5	39	45
								
PB20	8	BF	66	21.4	2.6	8.3	65	83
	8	NBF	67	15.0	1.8	5.2	45	52
	16	BF	53	18.0	2.2	6.9	54	69
	16	NBF	52	11.6	1.4	4.0	35	40
								
PB65	8	BF	53	17.7	2.1	6.8	53	68
	8	NBF	54	12.2	1.5	4.2	37	42
	16	BF	33	11.5	1.4	4.4	35	44
	16	NBF	31	6.9	0.8	2.4	21	24

^a^ NPB, non‐parboiled grain; PB20, soaked at 20 °C for 24 h; PB65, soaked at 65 °C for 4 h.

^b^ Based on brown rice zinc content of 33 μg g^−1^ for biofortified rice (BF) and 21 μg g^−1^ for non‐biofortified rice (NBF).

^c^ Cooking zinc retention = 90%. Rice intake = 134 g for children 4–6 years and 422 g for women. Estimated average daily zinc requirement = 10 mg for pregnant women and 4 mg for children 4–8 years.

Furthermore, considering that in some countries farmers typically prefer under‐milled rice to be consumed at the household level whereas over‐milled rice is allocated for selling, biofortified rice consumed by households that produce their own parboiled rice could have a higher contribution to the zinc daily intake than biofortified rice purchased in markets. The contribution of biofortified zinc rice to daily zinc EAR% of parboiled rice produced at the household level could be double compared to rice processed in medium‐ and large‐scale parboiling facilities.

## CONCLUSIONS

Zinc from the endosperm moved to the outer layers during parboiling, especially when rough rice was soaked in hot water. For this reason, losses of zinc in milled rice were highest when rice was parboiled using conditions resembling those used in large commercial parboiling facilities. Rough rice soaked at room temperature during the parboiling process, as done in households and medium‐scale commercial facilities, could contribute more to daily zinc intake compared to rough rice soaked in hot water commonly used in large scale parboiling facilities. Biofortified rice could improve daily zinc intake of populations at risk of inadequate zinc intake that use rice as their main staple food; however, the benefit of biofortified rice will be reduced if rough rice is parboiled and over‐milled, particularly if soaked at high temperature. The production of biofortified rice varieties with higher zinc concentration in brown rice than their current levels should be considered for populations that consume mostly parboiled rice or highly milled rice.

## Supporting information


**Figure S1**. Summary of the parboiling process for rice samples of variety BRRI dhan28 used to evaluate the effect of water types (SOFT and HARD) and parboiling methods (LIM, BOIL, COM1 and COM2) on zinc and iron retention. Each parboiling method was repeated three times.

**Figure S2**. Summary of the parboiling process to evaluate the effect of water soaking temperature (20 °C and 65 °C) on zinc and iron true retention of three rice varieties (BRRI dhan28, BRRI dhan42 and BRRI dhan64) milled at different degrees of milling. Each parboiling and milling process for each variety was repeated three times.
