# Supplementary material for: Effect of parboiling conditions on zinc and iron retention in biofortified and non‐biofortified milled rice
Source: J Sci Food Agric. 2021 Jul 5;102(2):514–22. doi: 10.1002/jsfa.11379 (PMC9290027; doi:10.1002/jsfa.11379)
Supplement: Supplementary file 1 — Figure S1. Summary of the parboiling process for rice samples of variety BRRI dhan28 used to evaluate the effect of water types (SOFT and HARD) and parboiling methods (LIM, BOIL, COM1 and COM2) on zinc and iron retention. Each parboiling method was repeated three times. [file JSFA-102-514-s001.docx]

Rough rice variety BRRI dhan28

Parboiled SOFTCOM1 and HARDCOM1

Brown rice

Parboiled milled rice at 7.5% DOM SOFTCOM1DOM7.5 and HARDCOM1DOM7.5

Soaked rough rice with 31% moisture content

Soaking 600 g at 20 ºC, 24 h in SOFT or HARD

Parboiled SOFTBOIL and HARDBOIL

Parboiled milled rice

at 7.5% DOM SOFTBOILDOM7.5 and HARDBOILDOM7.5

Parboiled SOFTLIM and HARDLIM

Steaming 7 min, sundrying 2–3 days, 12–13% MC

Parboiled brown rice SOFTLIMDOM0 and HARDLIMDOM0

Parboiled milled rice

at 7.5% DOM SOFTLIMDOM7.5 and HARDLIMDOM7.5

Soaked rough rice with 31% moisture content

Soaking 200 g at 65 ºC, 4 h in SOFT or HARD

Parboiled SOFTCOM2 and HARDCOM2

Dehulling

Parboiled milled rice at

7.5% DOM SOFTCOM2DOM7.5 and HARDCOM2DOM7.5

Parboiled brown rice SOFTBOILDOM0 and HARDBOILDOM0

Parboiled brown rice SOFTCOM1DOM0 and HARDCOM1DOM0

Parboiled brown rice SOFTCOM2DOM0 and HARDCOM2DOM0

Milling (28.5 g at 7.5±0.5% DOM)

Dehulling 300 g of rough rice

Dehulling

Dehulling

Dehulling

Boiling 7 min, sundrying 2–3 days, 12–13% MC

Steaming 14 min, sundrying 2–3 days, 12–13% MC

Steaming 14 min, sundrying 2–3 days, 12–13% MC

Milling (28.5 g at 7.5±0.5% DOM)

Milling (28.5 g at 7.5±0.5% DOM)

Milling (28.5 g at 7.5±0.5% DOM)

**Supplementary Figure 1**. Summary of the parboiling process for rice samples of variety BRRI dhan28 used to evaluate the effect of water types (SOFT and HARD) and parboiling methods (LIM, BOIL, COM1 and COM2) on zinc and iron retention. Each parboiling method was repeated three times.
