# Supplementary material for: Effect of parboiling conditions on zinc and iron retention in biofortified and non‐biofortified milled rice
Source: J Sci Food Agric. 2021 Jul 5;102(2):514–22. doi: 10.1002/jsfa.11379 (PMC9290027; doi:10.1002/jsfa.11379)
Supplement: Supplementary file 2 — Figure S2. Summary of the parboiling process to evaluate the effect of water soaking temperature (20 °C and 65 °C) on zinc and iron true retention of three rice varieties (BRRI dhan28, BRRI dhan42 and BRRI dhan64) milled at different degrees of milling. Each parboiling and milling process for each variety was repeated three times. [file JSFA-102-514-s002.docx]

Rough rice of varieties BRRI dhan28, BRRI dhan42 and BRRI dhan64

Soaked paddy at 31% MC (S65)

Soaking 3400 g at 65 ºC, 4 h

Parboiled rough rice (PB65)

Steaming 14 min, sundrying 2–3 days, 12–13% MC

Parboiled brown rice (PB65DOM0)

Dehulling

Brown rice (NPB)

(NPBDOM0), n= 9

Non-parboiled milled rice with 1% DOM (NPBDOM1)

Parboiled milled rice with 1% DOM (PB65DOM1)

Dehulling

Soaked paddy at 31% MC (S20)

Soaking 3400 g, at 20 ºC, 24 h

Parboiled rough rice (PB20)

Steaming 14 min, sundrying 2–3 days, 12–13% MC

Parboiled brown rice (PB20DOM0)

Dehulling

Parboiled milled rice with 1% DOM (PB20DOM1)

Milling (28.5 g for batch at 1% DOM +/- 0.5%)

Milling (28.5 g for batch at 1% DOM +/- 0.5%)

Milling (28.5 g for batch at 1% DOM +/- 0.5%)

Analyzed Non-parboiled milled rice with 1% DOM

Analyzed parboiled milled rice with 1% DOM

Analyzed parboiled milled rice with 1% DOM

Zinc and iron analysis

Zinc and iron analysis

Zinc and iron analysis

Non-parboiled milled rice with 2% DOM (NPBDOM2)

Parboiled milled rice with 2% DOM (PB65DOM2)

Parboiled milled rice with 2% DOM (PB20DOM2)

Milling to 1% DOM higher

Milling to 1% DOM higher

Milling to 1% DOM higher

Analyzed non-parboiled milled rice with 2% DOM. Previous two steps repeated 14 times to get NPBDOM3–NPBDOM16

Analyzed parboiled milled rice with 2% DOM. Repeat previous two steps 14 times to get PB65DOM3–PB65DOM16

Analyzed parboiled milled rice with 2% DOM. Repeat previous two steps 14 times to get PB20DOM3–PB20DOM16

Zinc and iron analysis

Zinc and iron analysis

Zinc and iron analysis

**Supplementary Figure 2**. Summary of the parboiling process to evaluate the effect of water soaking temperature (20 ºC and 65 ºC) on zinc and iron true retention of three rice varieties (BRRI dhan28, BRRI dhan42 and BRRI dhan64) milled at different degrees of milling. Each parboiling and milling process for each variety was repeated three times.
